# The association between social capital and loneliness in different age groups: a population-based study in Western Finland

**DOI:** 10.1186/s12889-016-3248-x

**Published:** 2016-07-11

**Authors:** Fredrica Nyqvist, Christina R. Victor, Anna K. Forsman, Mima Cattan

**Affiliations:** Åbo Akademi University, Faculty of Education and Welfare Studies, Study Programme of Social Sciences, Social Policy, PB 311, 65101 Vaasa, Finland; College of Health and Life Sciences, Brunel University London, Uxbridge, Middlesex UB8 3PH UK; Åbo Akademi University, Faculty of Education and Welfare Studies, Study Programme of Social Sciences, Developmental Psychology, Vaasa, Finland; Faculty of Health and Social Sciences, Northumbria University, Newcastle Upon Tyne, UK

**Keywords:** Loneliness, Social capital, Age groups, Population-based survey, Western Finland

## Abstract

**Background:**

Previous studies of loneliness have largely focused on establishing risk factors in specific age groups such as in later life or in young people. Researchers have paid less attention to the link between social capital and loneliness across different age groups. The aim of this study was to examine the association between social capital and experienced loneliness in different age groups in a Finnish setting.

**Methods:**

The data originates from a population-based cross-sectional survey conducted among 4618 people aged 15–80 in Western Finland in 2011. The response rate was 46.2 %. The association between social capital, measured by frequency of social contacts, participation in organisational activities, trust and sense of belonging to the neighbourhood and loneliness was tested by logistic regression analyses stratified by four age groups.

**Results:**

Frequent loneliness (defined as experienced often or sometimes) was higher among younger people (39.5 %) compared to older people (27.3 %). Low levels of trust were linked to loneliness in all four age groups. The association between other aspects of social capital and loneliness varied across age groups.

**Conclusions:**

Frequent loneliness is common among the general adult population and could be seen as a public health issue. Our findings imply that low social capital, especially in terms of low trust, may be a risk factor for loneliness. However, further research is needed to assess the influence of poor health and reverse causality as explanations for the findings.

## Background

Loneliness is defined by Perlman and Peplau [1] as a subjective, unpleasant, and distressing phenomenon resulting from a discrepancy between an individual’s desired and achieved levels of social relations. Loneliness could, therefore, be said to arise from the perception of a mismatch between one’s desired level and/or quality of relationships and their actual level or quality. Loneliness, or the absence of loneliness, is commonly used in association with measures of mental health, especially among older people, to denote mental well-being [2].

There is an extensive body of research identifying the individual ‘social risk factors’ for loneliness [3, 4]. Recently, the association between social capital, health and well-being has received attention and the concept of social capital has become important in health promotion and public health research [5, 6]. The growing interest in social capital research reflects a renewed interest in socio-environmental factors as determinants of health and denotes a shift in focus from micro level risk factors to broader contextual factors on neighbourhood or societal levels [6]. Previous studies of social capital have largely focussed upon relationships with mortality, physical and mental health [6], with less interest in examining differences across age groups [7, 8] or the relationship with mental well-being including loneliness, happiness and quality of life [9, 10].

There is a general agreement that social capital can be described as a social resource [11] and that it includes both collective and individual dimensions [5]. The latter approach as developed within the sociological tradition, applies the network perspective underlining different values for different individuals and the exchange of support within these networks [12, 13]. In contrast, the collective approach, as developed by Putnam, understands social capital as a public or collective good based on community activities. This approach, often referred to the social cohesion definition of social capital [5, 14], is commonly used as an explanation for health inequalities. Putnam [5] suggests that social participation and trust in others enhances interaction between people which is beneficial for individuals living in the neighbourhood, community or society. Although Putnam focuses mainly on the strength of social cohesion within the community, he also recognises that social capital has relevance for the achievement of individual goals such as well-being and health [5]. Putnam’s definition of social capital integrates structural aspects such as social participation and social contacts with cognitive aspects including interpersonal or social trust and sense of belongingness. Structural social capital describes the networks, relationships, and institutions that link people and groups together, whilst the cognitive dimension reflects the values, trust, confidence and norms that characterise these relationships. This study is based on the social cohesion approach as developed by Putnam and views social capital as a resource that is acquired through involvement in social activities, i.e. structural social capital, which may foster cognitive social capital including trust and sense of belonging.

Previous research has paid little attention to the association between social capital as a theoretical construct and loneliness across different age groups. Older people embedded in networks characterised by greater social capital, as illustrated by diversity in the membership of their network, reported lower levels of loneliness, compared to those embedded in less diverse network types (e.g. family, congruent, restricted networks) [15]. Perceptions of the local community, length of time living in the community, quantitative aspects of social support and social networks have not been associated with loneliness, an exception being perceived support [16]. Although the diversity of indicators of social capital used in the literature varies, and complicates the interpretation of the conflicting findings, there is little evidence supporting a relationship between social capital and loneliness among the general adult population (those aged 18 years and older).

Established social risk factors for loneliness encompass elements of social capital and previous research has revealed that interpersonal or social trust as well as sense of belonging are negatively associated with loneliness among both younger and older people [17–19]. In addition, for older people, living alone and widowhood are established social risk factors for loneliness [3, 4, 20]. Furthermore, previous research has shown that social interactions may serve different functions throughout the life-course [21–24]. Among young people the number of supportive peers in the network appears to be more important for loneliness than among older people, while satisfaction with support is central in older age [25]. The activity theory predicts that engagement in organisational activities and social relations supports well-being in older people [26], while for example the theory of socioemotional selectivity argues that with increasing age older people are more selective in their relations [27]. Consequently, older people have a tendency to be more satisfied with their current social networks as compared to younger age groups. This suggests that there may be differences across age groups as to how loneliness is conceptualised in relation to social support and social engagement. This has also been observed in studies focusing on social capital and quality of life [7] and self-rated health [28]. The results suggest that social capital, in terms of access to institutional resources [7] including for example opportunity for leisure activities, as well as neighbourhood social capital [28], are more beneficial for health and well-being among older people as compared to younger age groups. One explanation proposed for this observation is that older people are more dependent on resources in community contexts, because of lack of access to other social resources such as those linked to employment.

Across Europe, and in Finland specifically, there is a range of studies reporting loneliness in later life [e.g. 18, 29, 30]. Loneliness is not, however, exclusive to older people. Previous research also shows that loneliness is common among young people; 15–30 % experience some degree of loneliness in early adulthood, whereas people in middle age report lower levels of loneliness [29, 31]. Loneliness increases in old age, especially in the oldest old, with as many as 50 % experiencing serious or moderate loneliness [32]. Nonetheless, population-based studies looking at the relationship between loneliness across age groups are much less common [20, 29]. Yang and Victor [29] hypothesise in a cross-sectional study that loneliness and age may be related either positively (loneliness increases with age) or negatively (loneliness decreases with age) or non-linear. They demonstrate that both age-related and non-linear patterns of loneliness may be observed when making cross-national comparisons.

To date there are few loneliness studies using social capital as a theoretical framework. Importantly, age-related prevalence in loneliness does not necessarily mean age related similarities in the correlates of loneliness. In this study, we therefore link loneliness to social capital resources and we assess the relationship across different age groups in a Finnish setting.

## Methods

### Sample

In 2011, the Finnish National Institute for Health and Welfare (THL) distributed 10,000 postal questionnaires to a representative sample of citizens in Western Finland aged between 15 and 80 years. The cross-sectional survey response rate was 46.2 % (*n* = 4,618/10,000). This moderate response rate reflects both a general decline in population survey response rates, and also the nature of the survey focus – mental health – which is considered as a sensitive subject by many [20]. However, a higher response rate was noted for older people, women (52.7 % as compared to 39.6 % for men), Swedish-speaking Finns (54.6 % as compared to 46.1 % for Finnish-speakers). The age group with the highest response rate, 63.3 %, was people aged 61–70. For a detailed description on the methodology and sample see, Herberts et al. [33].

### Socio-demographic variables

Standard socio-demographic background variables collected were age, gender, marital status (single, divorced, widowed, cohabiting, married), basic education (measured with an ordinal level variable ranging from 1 (elementary school) to 4 (matriculation) and language (Finnish, Swedish and other). For the analyses marital status was collapsed into two groups “married, cohabiting”, and “single, divorced, widowed”, and educational level into “higher” (matriculation) and “lower” (elementary, middle school, comprehensive school). We used corresponding age categories (15–29 years, 30–49 years, 50–64 years, 65–80 years) as in previous research to facilitate comparisons [34]. The language groups (Finnish, Swedish, other) were collapsed into two groups “Swedish” and Finnish, other”.

### Social capital variables

We included measures of both structural and cognitive social capital. Structural social capital was measured by frequency of social contacts and participation in social activities. Social contacts were measured by two statements: How often are you in contact with friends and neighbours, respectively? The response categories were “several times a week”, “several times a month”, “few times a year”, “never”, and “does not exist”. For the analysis of social contacts, the response categories “several times a week” and “several times a month” were combined and coded as “frequent social contact” and “few times a year”, “never”, and “does not exist” were combined and coded as “infrequent social contact”. Organisational activities were assessed with a single question: “How active are you when it comes to association activities?”, with the response alternatives “very active”, “fairly active”, “not very active”, and “not active at all”. This variable was dichotomized with the first two response alternatives as “active” and the latter two as “non-active”. Cognitive social capital was assessed by trust and a sense of belonging. Trust was assessed with one statement: “It is better not to trust anyone”. The statement was graded on four point Likert scale range ranging from “fully correct” to “fully incorrect”. Combining “fully correct” and “quite correct” into one category to indicate mistrust and “quite incorrect” and “fully incorrect” to indicate trust dichotomized the measure.

Sense of belonging was assessed with the statement: “I feel I belong and am part of my neighbourhood”. Similarly to the trust statement, the response alternative was graded on four point Likert scale ranging from “fully correct” to “fully incorrect”. Those who answered “fully correct” and “quite correct” were combined to indicate “strong” sense of belonging, and those who answered “quite incorrect” and “fully incorrect” to indicate “weak”. Thus we have three measures of structural social capital and two of cognitive social capital.

### Outcome

Our outcome variable, loneliness, was operationalised by the question “Do you feel lonely?” with four response alternatives: often, sometimes, seldom, never. In line with other studies, we dichotomized the variable (lonely, not lonely) by combining often and sometimes into one category and seldom and never into the other [e.g. 35]. However, we also undertook a descriptive analysis using all four categories.

### Analyses

Our analytic strategy consisted first of examining the distribution of loneliness and social capital by age groups. Pearson’s chi-square tests were conducted to analyse variations in loneliness according to social capital. Logistic regression was used to analyse interaction terms with age, as a continuous variable, and social capital in order to ascertain whether the association between social capital and loneliness varied by age. Age-stratified logistic regression analysis was then conducted to assess the relationship between social capital and loneliness while controlling for socio-demographic variables. The results were presented as odds ratios (ORs) and their 95 % confidence intervals (CIs). SPSS version 19 was used for the analyses.

## Results

Table 1 describes the characteristics of the study group and the distribution of the social capital and loneliness variables. We see that, although, frequent contact with friends was in general high, there were fewer people who reported frequent contacts in the older age groups, whilst the proportion of those reporting frequent contact with neighbours was highest in the oldest age group. Levels of organisational activity were reported by a minority of the participants but were highest in the older age group (65–80 years). Both trust and belonging were high across all age groups with the older age groups having the highest sense of belonging and the younger age groups the higher levels of trust. We see that frequent loneliness (often, sometimes) was highest among young adults and the proportion was lower in older age groups.

**Table 1:** Socio-demographic characteristics, social capital and loneliness for four different age groups in Western Finland, 2011

	15–29	30–49	50–64	65–80
	(*n* = 774)	(*n* = 1217)	(*n* = 1528)	(*n* = 1080)
Gender				
Men	39.5	41.2	43.6	46.3
Women	60.5	58.7	56.4	53.7
Educational level				
Lower	52.9	51.2	74.3	89
Higher	47.1	48.8	25.7	11.0
Marital status				
Single	51.2	16.9	23.9	31.5
Couple	48.8	83.1	76.1	68.5
Language				
Finnish	82.5	84.5	84.7	80.4
Swedish	17.5	15.5	15.3	19.6
Social contacts				
Friends				
Infrequent	4.0	14.3	22.8	24.8
Frequent	96.0	85.7	77.2	75.2
Neigbours				
Infrequent	53.7	42.3	38	30.3
Frequent	46.3	57.7	62	69.7
Organisational activity				
Not Active	80.6	75.1	76.9	70
Active	19.4	24.9	23.1	30
Trust				
Mistrust	16.0	15.5	21.0	23.0
Trust	84.0	84.5	79.0	77.0
Neighbourhood belonging				
Strong	69.5	80.5	82	85.9
Weak	30.5	19.5	18	14.1
Loneliness				
Often	7.3	5.1	4.8	3.3
Sometimes	32.2	28.7	26.0	24.0
Seldom	42.9	40.3	40.0	38.4
Never	17.6	25.9	29.2	34.4

We next analysed variations in loneliness according to social capital. We report the results for the group who described themselves as lonely (sometimes or often) and their social capital for each age grouping. Significant differences (*p* < 0.05) in loneliness between respondents reporting frequent and infrequent social contacts with friends and neighbours, trust and neighbourhood belonging were found in all age groups. Furthermore, significant differences in loneliness were found between organisational active and not so active respondents among those aged 30–64 years, while in the youngest and oldest age groups they were not significant. Table 2 indicates that loneliness among respondents with infrequent social contacts and those reporting mistrust was particularly common in the younger age groups. For example, 61.2 % of those aged 15–29 years with low trust reported loneliness as compared to only 35.6. % of the respondents with high social trust.

**Table 2:** Prevalence of loneliness (sometimes, often) by age group and social capital variables in Western Finland, 2011

	15–29 (*n* = 304)	30–49 (*n* = 401)	50–64 (*n* = 455)	65–80 (*n* = 280)
Social contacts:				
Friends				
Frequent	38.1	31	26.9	24.9
Infrequent	74.2	50	43.5	34.8
*p*	<0.001	<0.001	<0.001	0.003
Neigbours				
Frequent	30.6	28.4	25.7	21.9
Infrequent	47.1	41	38.5	38.3
*p*	<0.001	<0.001	<0.001	<0.001
Organisational activity				
Active	37.2	25.7	25.1	25.7
Not active	39.9	36.4	32.6	28
*p*	0.55	0.001	0.008	0.460
Trust				
Trust	35.6	29	26.3	22.5
Mistrust	61.2	58.9	47.1	39.4
p	<0.001	<0.001	<0.001	<0.001
Neighbourhood belonging				
Strong	33.5	29.4	26.9	23.4
Weak	53.2	51.1	48.5	46.9
*p*	<0.001	<0.001	<0.001	<0.001

We justified the age stratified analyses of different age groups in our multivariate logistic regression models of loneliness by first testing an interaction between age, as a continuous variable, and social capital in relation to loneliness. The results showed that age could be seen as an effect modifier for social contacts with friends (OR 1.01, 95 % CI 1.00–1.01, *p* < 0.001) and neighbours (OR 1.01, 95 % CI 1.01–1.01, *p* < 0.001), for trust (OR 1.01, 95 % CI 1.01–1.02, *p* < 0.001) and neighbourhood belonging (OR 1.02, 95 % CI 1.01–1.02, *p* < 0.001), however, not for organisational activity (OR 1.00, 95 % CI 1.00–1.00, *p* = 0.570).

In Table 3 the results are stratified by four age groups. Mistrust was the only variable that was associated with loneliness across all age groups. Furthermore, loneliness was associated with infrequent social contacts with friends in the three younger age groups and contact with neighbours for the youngest and the oldest age groups. A weak sense of neighbourhood belonging was associated with loneliness in the age groups 15–29, 50–64 and 65–80. Social participation was linked with loneliness only for the age group 30–49 years.

**Table 3:** Multivariate logistic regression model on the probability of experiencing loneliness in Western Finland, 2011

	15–29 (*n* = 771)			30–49 (*n* = 1193)			50–64 (*n* = 1496)			65–80 (*n* = 1045)		
	OR		(95 % CI)	OR		(95 % CI)	OR		(95 % CI)	OR		(95 % CI)
Social contacts												
Friends												
Frequent	1.00			1.00			1.00			1.00		
Infrequent	3.82	**	(1.54–9.47)	2.28	***	(1.56–3.34)	1.99	***	(1.47–2.68)	1.31		(0.91–1.89)
Neigbours												
Frequent	1.00			1.00			1.00			1.00		
Infrequent	1.45	*	(1.02–2.09)	1.10		(0.82–1.48)	1.18		(0.89–1.57)	1.65	**	(1.16–2.35)
Organisational activity												
Active	1.00			1.00			1.00			1.00		
Not Active	1.10		(0.73–1.66)	1.42	*	(1.03–1.96)	1.31		(0.97–1.79)	1.06		(0.75–1.50)
Trust												
Trust	1.00			1.00			1.00			1.00		
Mistrust	1.99	**	(1.27–3.12)	2.85	***	(1.96–4.13)	1.77	***	(1.30–2.41)	1.61	*	(0.75–1.50)
Neighbourhood belonging												
Strong	1.00			1.00			1.00			1.00		
Weak	1.78	**	(1.23–2.59)	1.48		(0.95–1.97)	1.64	**	(1.17–2.30)	1.79	**	(1.15–2.78)

Note: Adjusted for gender, age (continuous), marital status, language, education

***p < 0.001; **p < 0.01; *p < 0.05

## Discussion

In this study, we assessed the prevalence of loneliness across a range of adult age groups in Western Finland and examined the association between social capital and experienced loneliness among a representative sample of persons aged 15–80. Five indicators of social capital, measuring structural and cognitive aspects of social capital were used. Our study demonstrated three main results. First, the prevalence of loneliness was negatively associated with age, i.e. it was higher among younger people as compared with older people. Second, low levels of trust were associated with loneliness in all the age groups. Third, both structural and cognitive elements of social capital were associated with loneliness. This may imply that the relationship between loneliness and social capital varies between age groups, which complicate the interpretation of the findings.

Similar to previous studies, we found that frequent loneliness (as experienced often or sometimes) is common among younger people [29, 31]. Older people reported the lowest levels of loneliness. This contrast with evidence from the European Social Survey showing that loneliness increased among people aged 60 and over, compared with younger people, in most European countries, including Finland [29]. However, a recent study of loneliness among the very old conducted in regions of western Finland and northern Sweden, showed that the levels of loneliness increased from the age of 85 so that those aged 95 years and over experienced the highest levels of loneliness [36]. This suggests that our study might have revealed a different pattern regarding loneliness among various age groups if we had included the ‘oldest old’.

We cannot determine if the observed differences in loneliness across ages reflect age or cohort effects, although the latter has largely been ruled out as an explanation to age differences in loneliness [3]. Victor et al. [37] and Tornstam [38] report that the prevalence of loneliness for older people has remained stable or even decreased on a population level during the past decades in Britain and Sweden, respectively. A previous study in Finland comparing the levels of loneliness between 1996 and 2006 reported that frequent levels of loneliness decreased slightly during the study period in all age groups, including older people aged 65–79 years, which the authors attributed to reduced unemployment and increased good self-rated health in these populations [34].

Social capital helps us to explain the interaction between environmental and individual factors and emphasises the importance of social resources across a range of contexts, such as neighbourhoods. Social capital has been found to be unequally distributed in the population [39], a feature present in our study (see Table 1). Previous research suggests that social capital may promote health and well-being [5, 6] and it has been hypothesized that it’s importance may increase as people age [7, 28], mainly due to retirement and loss of work related networks. A competing hypothesis posits that older people have the time to take part actively in organisational activities and to connect socially with friends and neighbours. This suggests that social capital in terms of neighbourhood cohesion may be of more importance for the prevention of loneliness in older people as compared with younger people. However, our results do not suggest a larger beneficial role of social capital in old age. For example, the effect size of the association between friend contacts and loneliness decreased clearly with age, so that in the oldest age group the association weakened and was rendered insignificant.

Infrequent contact with neighbours was associated with loneliness in both young and older adults. A plausible reason is that older people usually have lived a long time in the same place of residence and after retirement they are likely to spend more time in the neighbourhood as compared to other age groups [28]. For young adults, the neighbourhood environment may be a key socializing domain and provide an important context for well-being [40], especially if they still live in the place where they grew up. Sense of belonging to the neighbourhood may also capture neighbourhood attachment, or the degree to which one is included in various neighbourhood networks. Infrequent neighbourhood contacts and weak sense of belonging were both significantly related to higher levels of loneliness in the bivariate analyses (Table 2). However, in our multivariate analyses, the association for the age group 30–49 was no longer statistically significant. The association seems therefore to be indirect and mediated by other factors in the final model.

The relevance of social participation in terms of taking part in organisational activities for well-being has produced conflicting results [6, 10]. In our study organisational activities were associated with loneliness only among people in the age group 30–49. This may reflect that organisational activities in this age group might be a welcome alternative to hours spent in paid work and at home doing domestic work. Or, it may reflect that family and work situations among people engaged in activities in this age group vary compared to those who are not engaged. This is an issue that warrants further investigation with more attention given to gender differences.

A range of studies have reported beneficial health effects of experiencing trust [41, 42]. Our study demonstrated a strong association between low levels of trust and loneliness. Even if Putnam suggested that structural social capital in terms of social participation foster trust [5], alternative explanations have suggested that trust is an important characteristic necessary in order to be able to interact with other people and to develop supportive relations [43]. Previous research suggests that social trust is promoted when people feel included in society and are treated with justice and fairness [41], indicating that welfare state characteristics could also influence the levels of social capital.

It is important to be aware that although the relationship between social capital and loneliness seems partly related to age, it may also be a function of other influential contextual factors such as marital and employment status, which provide the individual with social connections and an increased opportunity to social interactions [20]. In addition, health status may be a confounder because of its association with both social capital and loneliness [2, 6]. These issues should be considered and more thoroughly studied in forthcoming studies analysing age differences in social capital and loneliness.

### Limitations of this study

Data used in this study were obtained through the population-based survey of mental health in Western Finland. The moderate response rate of 46.2 % might imply respondent-bias, considering the higher response rate for older people, women and Swedish-speaking Finns [33]. Further, the older people in the survey reported lower levels of loneliness as compared to previous studies, which might indicate a selection bias. In a Finnish context, it is also important to consider the regional differences in health [44], which might limit the generalisation of our findings in Western Finland to other areas.

The operationalisation of structural and cognitive social capital was limited although similar items have been used in previous studies [6, 10]. Dichotomization of variables implies a loss of information. However, by reducing the number of response categories of the social capital indicators we distinguished between differences in loneliness in groups of high and low social capital. We used a single-item self-report loneliness rating question instead of scales such as the Jong Gierveld Scale or the UCLA Loneliness Scale [2]. However, they are all regarded as valid measures of loneliness, as discussed by Victor et al. [37]. Finally, the cross-sectional nature of this study only allows the assessment of differences in inferential associations and not of causal relations between variables and do not enable us to differentiate between cohort or age effects. This is important considering that the composition of social capital and loneliness and the relationship between them are likely to differ through the life course [5, 45]. However, despite these limitations our study has produced some novel findings and offers several potential areas for further research.

## Conclusions

Loneliness is common among younger age groups and thus not only a problem of growing older. Access to different aspects of social capital has different associations with loneliness across age. Overall, access to friends, neighbourhood belonging and being able to social trust are linked to reduced loneliness. Our results imply that interventions that aim to increase social capital for health-promoting purposes need to be age specific. Nevertheless, more research is needed in order to examine the influence of poor health and reverse causality as explanations for our findings.

## Abbreviations

CI, Confidence Interval; OR, Odds Ratio
